# Prise en charge des envenimations par morsure de serpent à l’Institut de recherche en biologie appliquée de Guinée

**DOI:** 10.48327/mtsi.v6i1.2026.803

**Published:** 2026-01-26

**Authors:** Ousmane BALDÉ, Mamadou Alpha BALDÉ, Mamadou Cellou BALDÉ, Mohamed Ciré DIALLO, Mohamed Sahar TRAORÉ

**Affiliations:** 1 Clinique Asclepius Snakebite Foundation, Kindia, Guinée; 2 Institut de recherche en biologie appliquée de Guinée, Kindia, Guinée

**Keywords:** Envenimation, Traitement, Antivenin, Létalité, Viperidae, Elapidae, Kindia, Guinée, Afrique subsaharienne, Envenomation, Treatment, Antivenom, Mortality, Viperidae, Elapidae, Kindia, Guinea, Sub-Saharan Africa

## Abstract

**Objectifs:**

Le but de cette étude est de présenter les caractéristiques épidémiologiques et cliniques des envenimations par morsure de serpent traitées de 2015 à 2019 à Kindia, en Guinée.

**Matériel et méthodes:**

Nous avons réalisé une étude rétrospective descriptive d’une période de 5 ans (janvier 2015 à décembre 2019), incluant tous les dossiers de patients admis pour envenimation par morsure de serpent à l’Institut de recherche en biologie appliquée de Guinée, à Kindia.

**Résultats:**

Il a été enregistré 1 420 cas de morsure dont 1 008 envenimations (sex-ratio H/F = 1,2) en 60 mois. Les patients provenaient majoritairement du milieu rural (76,9 %) et pratiquaient principalement l’agriculture et l’élevage. Les morsures siégeaient aux membres inférieurs dans 85,4 % des cas. Le syndrome vipérin était dominant et 98,5 % de nos patients ont bénéficié d’un antivenin avec un taux de guérison de 95,9 %.

**Conclusion:**

Les résultats de cette étude montrent que les accidents d’envenimation par morsure de serpent sont fréquents et entraînent une létalité de 2,5 %. Les résultats plaident en faveur d’une meilleure prise en compte des morsures de serpents dans la politique sanitaire de la Guinée.

## Abstract (en)

**Objectives:**

This study aims to present the epidemiological and clinical characteristics of snakebites treated in Kindia, Guinea, between 2015 and 2019.

**Materials and methods:**

We conducted a retrospective descriptive study covering the period from January 2015 to December 2019. The study included all patient records for envenomation from snakebites at the Institut de Recherche en Biologie Appliquée de Guinée in Kindia.

**Results:**

A total of 1,420 snakebites were recorded, including 1,008 envenomations (male-to-female ratio = 1.2), over a 60-month period. Most patients (76.9%) came from rural areas and were primarily involved in agriculture and livestock farming. In 85.4% of cases, the bites were to the lower limbs. Viper syndrome was predominant, and 98.5% of patients received antivenom with a 95.9% cure rate.

**Conclusion:**

The results of this study demonstrate that snakebite envenomation are common and have a 2.5% case-fatality rate. These results suggest the need for greater consideration of snakebites in Guinea’s health policy.

## Introduction

Les envenimations ophidiennes touchent particulièrement les régions tropicales du monde où les formations végétales sont variées et où les activités agropastorales dominent [2]. En Afrique subsaharienne, plus de 310 000 patients sont traités chaque année dans les centres de santé pour une envenimation ophidienne, entraînant près de 7 500 décès (2,4 %) et au moins autant de handicaps locomoteurs [3]. En Guinée, il n’existe pas de statistique précise sur l’incidence et la gravité des envenimations par morsure de serpent. Cependant, une enquête auprès des ménages et des tradipraticiens a montré que l’incidence annuelle des morsures de serpent était de 375 pour 100 000 habitants avec une mortalité de 19,2 pour 100 000 habitants [1]. Les problèmes rencontrés dans la prise en charge précoce des victimes dans les pays en voie de développement sont liés aux difficultés d’accès aux centres de soins, au manque de formation du personnel soignant, à la non-disponibilité d’un antivenin (AV), elle-même liée à son coût élevé par rapport aux ressources de la population [3,6].

Depuis les années 1990, l’Institut de recherche en biologie appliquée de Guinée (IRBAG) traite plusieurs centaines de cas annuels survenant dans la région de Kindia avec une létalité moyenne de 2,2 % et un taux d’amputation de 2,1 % lorsqu’un AV était disponible; en l’absence d’AV, la létalité peut dépasser 18 % [2]. Nous présentons les caractéristiques épidémiologiques d’une série d’envenimations par morsure de serpent traitées à l’IRBAG de 2015 à 2019.

## Matériel et méthodes

**Figure 1:**
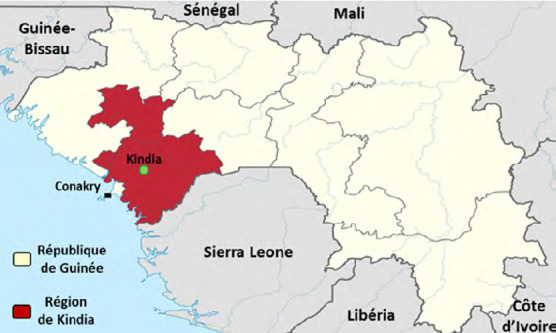
République de Guinée, localisation de la région de Kindia

Kindia est située à 135 km au nord-est de Conakry, la capitale de la Guinée (Fig. 1). De tradition agro-pastorale, la commune urbaine de Kindia s’étend sur 500 km^2^ et compte 182 280 habitants [7].

Le relief est constitué de plateaux, de plaines et des montagnes dont le point culminant est le mont Gangan (1 117 m).

La région de Kindia est dotée d’un climat tropical humide avec une végétation constituée de savanes arborées et de forêts.

Il s’agit d’une enquête rétrospective descriptive menée à l’IRBAG à partir des dossiers médicaux, du registre de consultation des patients et de la fiche d’enquête qui ont servi de support pour la collecte des données.

L’IRBAG est un établissement public à caractère scientifique et technique placé sous la tutelle du ministère chargé de la recherche scientifique. Il a pour mission la promotion et le développement des activités de recherche en biologie médicale dans les domaines fondamentaux et appliqués. Les missions de son département de venimologie sont l’étude des animaux venimeux, le prélèvement des venins de serpents, la formation et la prise en charge des cas de morsures de serpents. À ce titre, un dispensaire spécialisé dans le traitement des envenimations ophidiennes existe depuis les années 1990 [2]. Ce centre de santé comporte une salle de consultation où sont pratiqués les soins délivrés aux patients mordus par un serpent et deux salles d’hospitalisation de cinq lits au total. Le personnel de la clinique est composé de deux médecins, trois infirmiers, un laborantin et deux aides-soignants hygiénistes.

Cette étude porte sur l’ensemble des dossiers des patients qui ont été admis du 1^er^ janvier 2015 au 31 décembre 2019 pour morsure de serpent. Un patient était considéré comme envenimé lorsqu’il présentait un ou plusieurs symptômes résultant de l’action du venin d’un serpent [4]. Les variables recueillies concernaient la démographie (âge, genre, profession, lieu, date, saison et siège de la morsure), la symptomatologie à l’admission, le traitement et l’évolution pendant l’hospitalisation. La symptomatologie a permis de distinguer les envenimations par Viperidae de celles par Elapidae. Une envenimation vipérine associe : saignement, douleur, œdème et parfois nécrose. Une envenimation par Elapidae est, dans un premier temps, essentiellement sensorielle (anesthésie, picotement, fourmillements, tremblement musculaire au niveau du membre mordu, puis troubles de l’audition et de la vision) peu accessibles à l’examen. Le premier symptôme nettement objectif est la ptose palpébrale et symétrique.

Lorsque les informations sur le délai entre la morsure et l’arrivée à la clinique étaient incomplètes, les dossiers n’ont pas été inclus.

Les données ont été collectées à l’aide d’une fiche d’enquête préétablie à travers l’application Kobocollect dans sa version v1.30.1 (KoboToolbox; https://www.kobotoolbox.org/). Elles ont été traitées et analysées avec le logiciel SPSS (IBM SPSS; version 21.0).

## Résultats

Du 1^er^ janvier 2015 au 31 décembre 2019, nous avons enregistré 1 420 cas de morsures de serpent dont 1 008 (71 %) présentaient une envenimation. Les 412 morsures asymptomatiques (29 %) étaient dues à des espèces venimeuses n’ayant pas inoculé de venin ou à des serpents non venimeux. Il a été remarqué un pic des envenimations en 2018 avec 268 cas tandis que le nombre de cas le plus bas a été observé en 2019 avec 128 cas. La majorité des morsures est survenue en saison des pluies (mai à octobre : n = 748; 74,2 %) contre 260 envenimations (25,8 %) pendant les 6 mois de saison sèche. La provenance des patients était diverse : 775 (76,9 %) de la région administrative de Kindia et 233 (23,1 %) des autres régions de Guinée (Conakry, Boké, Mamou, Pita, Faranah, Kankan et N’Nzérékoré) (Tableau I).

**Tableau I:** Caractéristiques démographiques

Variable	Effectif (n = 1 008)	%
Genre		
homme	554	55
femme	454	45
Tranche d’âge
1-10 ans	98	9.7
11-20	309	30.7
21-30	188	18.7
31-40	157	15.6
41-50	105	10.4
51-60	84	8.3
> 60	67	6.6
Profession		
ménagère	353	35
élève-étudiant	240	23,8
cultivateur	202	20
éleveur	44	4,4
profession libérale	90	9
sans emploi	79	7,8

Un syndrome inflammatoire local, accompagné ou non par des troubles de la coagulation, a été observé chez 848 patients (84,1 %). Des troubles neurologiques ont été retrouvés chez 160 patients (15,9 %) (Tableau II).

**Tableau II:** Symptomatologie clinique

Variable	Effectif (n = 1 008)	%
Siège de la morsure		
membres inférieurs	861	85,4
membres supérieurs	142	14,1
tête	2	0,2
tronc	2	0,2
non défini	1	0,1
Signes cliniques		
douleur	813	80,7
œdème	651	64,6
saignement local	608	60,3
asthénie	187	18,6
vomissement	171	17
épigastralgie	88	8,7
troubles visuels (ptosis)	84	8,3
paralysie nasopharyngée	41	4,1
nécrose	19	1,9
dyspnée-paralysie respiratoire	8	0,8

Le traitement est précisé dans le Tableau III. L’AV utilisé a été l’Inoserp^TM^ PAN-AFRICA fabriqué par Inosan Biopharma (Mexique) (plusieurs lots différents, la plupart dosés à 500 LD_50_ et les autres à 250 LD_50_ par ampoule). Pour les espèces venimeuses d’Afrique subsaharienne, la posologie était celle recommandée par le fabricant qui tient compte de la gravité de l’envenimation.

**Tableau III:** Traitement

Traitement	Effectif (n = 1 008)	%
Antivenin	994	98,5
Antibiothérapie	825	81,8
Antalgique-Anti-inflammatoire	544	54
Réhydratation par Ringer Lactate	127	12,6

L’antibiothérapie était composée de l’amoxicilline 500 mg et du métronidazole 250 mg.

Seize patients présentant une nécrose ou une autre complication, ont été référés à l’hôpital régional de Kindia. Tous avaient reçu de l’AV avant d’être référés. Parmi les 25 patients décédés, 14 avaient un syndrome neurotoxique (paralysie respiratoire).

## Discussion

Cette étude rétrospective présente comme principale limite les faibles moyens diagnostiques et thérapeutiques de l’IRBAG faute de ressources appropriées. De plus, l’étude rétrospective ne permet pas le recueil de toutes les données pertinentes, comme l’identification du serpent, le retard de consultation, les traitements traditionnels reçus par la victime avant son admission à la clinique de l’IRBAG.

Dépourvu de laboratoire et d’appareil de réanimation, le centre de traitement des envenimations de l’IRBAG est représentatif d’un dispensaire périphérique d’Afrique subsaharienne. En revanche, son personnel bénéficie d’une ancienne et solide expérience de la prise en charge des envenimations ophidiennes, ce qui permet d’évaluer l’apport de techniques et de traitements simples, incluant depuis la fin des années 2000 un approvisionnement régulier et suffisant d’AV efficaces et bien tolérés (Antivipmyn^TM^ Africa de 2007 à 2015, puis Inoserp^TM^ PAN-AFRICA).

La variation saisonnière de l’incidence s’explique à la fois par l’activité humaine (notamment agricole) et les rythmes biologiques des serpents (accouplements et pontes ou mises bas) [5].

Le profil démographique, le siège de la morsure et la symptomatologie des patients traités à l’IRBAG sont similaires à ceux décrits dans la littérature [3]. La prépondérance des envenimations vipérines est liée à l’abondance des Viperidae en Afrique subsaharienne. Il est probable que l’évolution plus rapide vers le décès après une morsure par Elapidae ne permette pas d’atteindre le centre de traitement. Il faut toutefois souligner que la fréquence des signes neurologiques est en faveur d’une implication fréquente des Elapidés [3]. Ces derniers sont probablement plus abondants dans la région de Kindia qu’ailleurs.

Dans notre série, la létalité est semblable à celle que l’on observe dans les hôpitaux d’Afrique subsaharienne [3]. Les envenimations vipérines, plus fréquentes, ont un taux de létalité plus faible que les envenimations par Elapidae. La rapidité d’action du venin et le délai de consultation parfois important expliquent une létalité plus élevée après une morsure par Elapidae. En outre, le délai de consultation dépend de deux facteurs essentiels qui expliquent en grande partie l’évolution défavorable de l’envenimation (nécrose et/ou décès) : l’origine parfois éloignée du patient et son passage chez le tradipraticien avant de consulter dans un centre de santé.

Malgré les moyens modestes de l’IRBAG, les résultats obtenus montrent que l’utilisation d’un SAV permet de prendre en charge la plupart des victimes d’envenimation ophidienne dans un centre de santé modeste. Le recours à un hôpital disposant d’un plateau technique sophistiqué peut ainsi être réservé aux patients présentant une complication qui sont minoritaires.

Cela renforce la stratégie qui consiste à mettre le SAV au plus près des populations les plus exposées, notamment dans les centres de santé périphériques [6].

## Conclusion

Cette étude présente les caractéristiques épidé-miologiques et cliniques des patients admis pour envenimation à l’IRBAG. Elles sont similaires à celles décrites dans d’autres travaux en Afrique subsaharienne, confirmant la prédominance des syndromes vipérins, la fréquence élevée des morsures au niveau des membres inférieurs et la survenue majoritaire en milieu rural et en saison pluvieuse. L’utilisation de l’immunothérapie a permis d’obtenir un taux de guérison élevé de 95,9 % et un taux de létalité relativement faible (2,5 %). L’accessibilité de l’immunothérapie au plus près des victimes et la formation du personnel de santé à la prise en charge des envenimations ophidiennes constituent des facteurs clés pour réduire durablement la mortalité et les séquelles liées aux morsures de serpent en Guinée.

## Déclaration institutionnelle

Le protocole de cette étude a été approuvé par le comité d’éthique institutionnel de l’Institut de recherche en biologie appliquée de Guinée (IRBAG) (N°32/CEI/IRBAG) du 7 novembre 2022.

## Financement de l’étude

Cette étude n’a reçu aucun financement.

## Contribution des auteurs

Conception et réalisation de l’étude : BO, BMA et BMC.

Collecte des données : BO, BMC et DMC.

Analyse et interprétation des données : BO, TMS et BMA.

Recherche documentaire : BO et BMA.

Rédaction du manuscrit : BO et BMA.

Révision du manuscrit : BMC et TMS.

Tous les auteurs ont lu et approuvé la version finale du manuscrit.

## Déclaration de liens d’intérêts

Les auteurs déclarent ne pas avoir de conflit d’intérêt.
