# Supplementary material for: Treatment of snakebites at the Guinea Institute for Applied Biology Research
Source: Med Trop Sante Int. 2026 Jan 26;6(1):mtsi.v6i1.2026.803. [Article in French] doi: 10.48327/mtsi.v6i1.2026.803 (PMC13216904; doi:10.48327/mtsi.v6i1.2026.803)
Supplement: Related texts

# Research article: Treatment of snakebites at the Guinea Institute for Applied Biology Research

## Introduction

Snakebite envenomations are particularly common in tropical regions of the world with a wide variety of vegetation where agriculture and animal husbandry are the main activities [2]. In sub-Saharan Africa, over 310,000 people receive treatment for snakebites at health centers each year, resulting in nearly 7,500 deaths (2.4%) and an equal number of cases of locomotor disability [3]. In Guinea, there are no precise statistics on the incidence and severity of snakebites. However, a survey of households and traditional healers revealed an annual incidence rate of 375 bites per 100,000 people, with a mortality rate of 19.2 per 100,000 people [1]. The issues encountered in the early treatment of victims in developing countries are linked to difficulties accessing healthcare centers, a lack of healthcare personnel training, and the unavailability of antivenom (AV), which is linked to its high-cost relative to the population’s resources [3,6].

Since the 1990s, the Institut de Recherche en Biologie Appliquée de Guinée (IRBAG) has treated several hundred cases annually in the Kindia region. With AV available, the average case-fatality rate was 2.2%, and the amputation rate was 2.1%. Without AV, the case fatality rate can exceed 18% [2]. Here, we present the epidemiological characteristics of a series of snakebite envenomations treated at IRBAG from 2015 to 2019.

## Materials and methods

Kindia is located 135 kilometers northeast of Conakry, the capital of Guinea (Fig. 1). The traditionally agropastoral urban commune covers 500 km² and has a population of 182,280 [7].

**Figure 1:** Republic of Guinea, location of the Kindia region

Source: Profoss et NordNordWest. 2011. Kindia in Guinea. Wikimedia Commons. Creative Commons Attribution-ShareAlike. https://commons.wikimedia.org/wiki/File:Kindia_in_Guinea.svg?lang=fr

Its terrain consists of plateaus, plains, and mountains; the highest point is Mount Gangan, which reaches 1,117 meters.

The Kindia region has a humid tropical climate, and its vegetation consists of wooded savannas and forests.

This retrospective descriptive study was conducted at IRBAG based on medical records, patient consultation registers, and survey forms used to collect data.

IRBAG is a public scientific and technical institution under the supervision of the Ministry of Scientific Research. Its mission is to promote and develop medical biology research in fundamental and applied fields. The venomology department studies venomous animals, collects snake venom, provides training, and treats snakebites. A clinic specializing in the treatment of snakebites has existed since the 1990s [2]. The clinic has a consultation room for treating patients bitten by snakes and two hospital rooms with a total of five beds. The clinic’s staff consists of two doctors, three nurses, one laboratory technician, and two nursing assistants.

This study covers all patient records of individuals admitted for snakebites between January 1, 2015, and December 31, 2019. A patient was considered poisoned if they presented with one or more symptoms resulting from snake venom action [4]. The collected variables concerned demographics (e.g., age, gender, occupation, location, date, season, and site of the bite), symptoms upon admission, treatment, and progress during hospitalization. These symptoms allowed us to distinguish between envenomation by Viperidae and Elapidae. Viperine envenomation is associated with bleeding, pain, edema, and sometimes necrosis. Elapidae envenomation initially presents mainly as sensory symptoms (numbness, tingling, and a pins-and-needles sensation), followed by muscle tremors in the bitten limb and problems with hearing and vision. These symptoms are not easily detectable on examination. The first clearly objective symptom is ptosis of the eyelids on both sides of the face.

Files with incomplete information on the time between the bite and arrival at the clinic were excluded.

Data were collected using a pre-established survey form via the Kobocollect application (version v1.30.1; KoboToolbox; https://www.kobotoolbox. org/). The data were processed and analyzed using IBM SPSS Statistics version 21.

## Results

From January 1, 2015, to December 31, 2019, we recorded 1,420 snakebite cases, 1,008 (71%) of which were venomous. The 412 asymptomatic bites (29%) were caused by either venomous snakes that did not inject venom or nonvenomous snakes. The highest number of envenomations occurred in 2018 with 268 cases, while the lowest number occurred in 2019 with 128 cases. Most snake-bites occurred during the rainy season (May to October) with 748 cases (74.2%), compared to 260 cases (25.8%) during the dry season. The patients came from various locations: 775 patients (76.9%) were from the Kindia administrative region, and 233 patients (23.1%) were from other regions of Guinea (Conakry, Boké, Mamou, Pita, Faranah, Kankan, and N’Zérékoré) (Table I).

**Table I:** Demographic characteristics

Variable / Variable	Effectif (n = 1 008) / Headcount (n = 1,008)	% / %
Genre / Gender		
>homme / male	554	55
>femme / female	454	45
Tranche d’âge / Age group
>1-10 ans / 1-10 years	98	9.7
>11-20 / 11-20 years	309	30.7
>21-30 / 21-30 years	188	18.7
>31-40 / 31-40 years	157	15.6
>41-50/ 41-50 years	105	10.4
>51-60 / 51-60 years	84	8.3
>> 60 / > 60	67	6.6
Profession / Occupation		
>ménagère / ousewife	353	35
>élève-étudiant / pupil/student	240	23,8
>cultivateur / farmer	202	20
>éleveur / livestock breeder	44	4,4
>profession libérale / liberal profession	90	9
>sans emploi / unemployed	79	7,8

A local inflammatory syndrome accompanied by coagulation disorders was observed in 848 patients (84.1%). Neurological disorders were found in 160 patients (15.9%) (Table II).

**Table II:** Clinical symptoms

Variable / Variable	Effectif (n = 1 008) / Headcount (n = 1,008)	% / %
Siège de la morsure / Location of bite		
>membres inférieurs / lower limbs	861	85,4
>membres supérieurs / upper limbs	142	14,1
>tête / head	2	0,2
>tronc / torso	2	0,2
>non défini / undefined	1	0,1
Signes cliniques / Clinical signs		
>douleur / pain	813	80,7
>œdème / edema	651	64,6
>saignement local / local bleeding	608	60,3
>asthénie / asthenia	187	18,6
>vomissement / vomiting	171	17
>épigastralgie / epigastric pain	88	8,7
>troubles visuels (ptosis) / visual disturbances (ptosis)	84	8,3
>paralysie nasopharyngée / nasopharyngeal paralysis	41	4,1
>nécrose / necrosis	19	1,9
>dyspnée-paralysie respiratoire / dyspnea/respiratory paralysis	8	0,8

The treatment is specified in Table III.

**Table III:** Treatmen

Traitement / Treatment	Effectif (n = 1 008) / Headcount (n = 1,008)	% / %
Antivenin / Antivenom	994	98,5
Antibiothérapie / Antibiotic therapy	825	81,8
Antalgique-Anti-inflammatoire / Analgesic-Anti-inflammatory	544	54
Réhydratation par Ringer Lactate / Rehydration with Ringer’s lactate	127	12,6

The AV used was Inoserp^TM^ PAN-AFRICA, manufactured by Inosan Biopharma (Mexico), in several different batches. Most were dosed at 500 LD_50_, and the rest at 250 LD_50_, per ampoule. For venomous species in sub-Saharan Africa, the recommended dosage by the manufacturer was used, taking into account the severity of the envenomation.

Antibiotic therapy consisted of 500 mg of amoxicillin and 250 mg of metronidazole.

Sixteen patients with necrosis or other complications were referred to Kindia Regional Hospital. All had received AV prior to referral. Of the 25 patients who died, 14 had neurotoxic syndrome (respiratory paralysis).

## Discussion

The main limitation of this retrospective study is IRBAG’s limited diagnostic and therapeutic resources, which are due to an absence of adequate facilities. Additionally, the retrospective nature of the study prevented the collection of all relevant data, including the identification of the snake, the delay in seeking medical attention, and the traditional treatments the victim received prior to admission to the IRBAG clinic.

Without laboratory or resuscitation equipment, the IRBAG venomous snake treatment center represents a typical peripheral health center in sub-Saharan Africa. However, the center’s staff has extensive experience in managing snakebites, enabling the evaluation of the effectiveness of simple techniques and treatments. Since the late 2000s, the center has had a regular and sufficient supply of effective and well-tolerated AVs, including Antivipmyn^TM^ Africa from 2007 to 2015 and Inoserp^TM^ PAN-AFRICA thereafter.

Seasonal variation in incidence can be explained by human activity, particularly agriculture, and the biological rhythms of snakes, such as mating, egg-laying, and birthing [5].

The demographic profile, location of the bite, and symptoms of patients treated at IRBAG are similar to those described in the literature [3]. The preponderance of viperine envenomations is linked to the abundance of Viperidae in sub-Saharan Africa. It is likely that the rapid progression of death after an Elapidae bite prevents patients from reaching the treatment center in time. However, the frequency of neurological signs suggests frequent involvement of Elapidae [3]. They are probably more abundant in the Kindia region than elsewhere.

In our study, the case-fatality rate was similar to that observed in hospitals in sub-Saharan Africa [3]. Viper bites are more common and have a lower case-fatality rate than Elapidae bites. The rapid action of the venom and the often lengthy delay in seeking medical attention explain the higher case-fatality rate following an Elapidae bite. The delay in seeking medical attention depends on two key factors that largely determine the outcome of envenomation: the patient’s remote location and their visit to a traditional healer before consulting a health center.

Despite its modest resources, IRBAG’s results show that most ophidian envenomation victims can be treated in a modest health center with an AAS. Thus, the use of a hospital with sophisticated technical facilities can be reserved for patients with complications, who are in the minority.

This reinforces the strategy of bringing the AAS as close as possible to populations at greatest risk, particularly in peripheral health centers [6].

## Conclusion

This study presents the epidemiological and clinical characteristics of patients admitted to IRBAG for envenomation. These characteristics are similar to those described in other studies in sub-Saharan Africa. However, our results confirm the predominance of viperine syndromes, the high frequency of snakebites to the lower limbs, and that the majority of cases occur in rural areas and during the rainy season. Immunotherapy resulted in a high recovery rate of 95.9% and a relatively low case-fatality rate of 2.5%.

Accessibility of immunotherapy and training of health personnel in ophidian envenomation management are key factors in reducing mortality and sequelae related to snakebites in Guinea.

## Institutional statement

The protocol for this study was approved by the Institutional Ethics Committee of the Guinea Institute for Applied Biology Research (IRBAG) (No. 32/CEI/IRBAG) on November 7, 2022.

## Funding

This study received no funding.

## Authors’ contributions

Study design and implementation: BO, BMA, and BMC.

Data collection: BO, BMC, and DMC.

Data analysis and interpretation: BO, TMS, and BMA.

Documentary research: BO and BMA.

Manuscript writing: BO and BMA.

Manuscript revision: BMC and TMS.

All authors have read and approved the final version of the manuscript.

## Conflicts of interest

The authors declare no conflicts of interest.
